# Is There any Association Between Total Laryngectomy and Sexual Disorders in Men?

**DOI:** 10.22038/IJORL.2022.61360.3109

**Published:** 2022-09

**Authors:** Kamyar Iravani, Leila Monshizadeh, Elmira Moeinjahromi, Amir Soltaniesmaeili, Ali Sahraian

**Affiliations:** 1 *Otolaryngology Research Center, Department of Otolaryngology, Shiraz University of Medical Sciences, Shiraz, Iran. *; 2 *Research Center for Psychiatry and Behavioral Sciences, Shiraz University of Medical Sciences, Shiraz- Iran. *

**Keywords:** Depression, Larynx, Sexual disorders, Surgery, Total laryngectomy

## Abstract

**Introduction:**

According to the prevalence of sexual enjoyment reduction in total or partial laryngectomy patients, the present study aimed to evaluate sexual disorders among men who had undergone total laryngectomy.

**Materials and Methods:**

In this cross-sectional case-control study, purposive sampling was carried out to select all the samples that had experienced total laryngectomy. The control group was selected among the male patients who were referred for a routine checkup. In order to compare the groups, the international index of erectile function (IIEF) was performed, and the data were statistically analyzed in SPSS software (version 21).

**Results:**

Based on the obtained results, laryngectomy patients had experienced problems with sexual problems, especially in the field of erectile function, sexual desire, and intercourse satisfaction (P<001).

**Conclusions:**

According to various studies, sexual dissatisfaction negatively impacts the Quality of life. This problem, commonly observed in total laryngectomy patients, needs to be considered.

## Introduction

Laryngeal cancer is one of the known malignancies in the head and neck anatomically divided into supraglottic, glottic, and subglottic cancers (1). According to the tumor size and various affected laryngeal parts, different interventions, such as hemilaryngectomy, total laryngectomy, chemotherapy, radiotherapy, or a combination of surgery and chemo-radiotherapy, will be performed (2). 

Hemilaryngectomy is recommended in the earlier cancer stages (T1 through large T2). In this surgery, some parts of the larynx and underlying thyroid cartilage will be removed (3). This procedure relatively maintains the three primary functions of the larynx, including breathing, swallowing, and phonating. 

Nonetheless, in the case of total laryngectomy recommended in advanced laryngeal cancer, the larynx is totally removed, and the airway is interrupted; therefore, respiration is performed through a tracheal stoma (4).

Finally, a complete separation of superior and inferior parts of the airway will occur, resulting in permanent loss of voice and smell, poor life quality, as well as difficulties in social interactions, self-esteem, and sexual behaviors (4,5). 

Apart from voice loss and swallowing difficulties of total laryngectomy discussed in many studies (6,7), sexual problems that might be indirectly caused by mood disorders (8) are a vital issue deserving special attention.

A study conducted in 2016 observed that depression and anxiety are the two most common causes that lead to difficulties in self-esteem and sexual functioning in patients with a total or partial laryngectomy (9). Reduced sexual enjoyment was also confirmed in another study carried out on patients who underwent laryngeal and hypopharyngial cancer surgery (10). 

This is mostly due to patients' post-operation depression that leads to loss of sound and speech ability. Although there are several studies in this filed, the vast majority of men, especially in Asian culture, do not tend to discuss this problem and, consequently, not to look for a solution.

 Therefore, the main focus of the present study is on sexual problems in men who had undergone total laryngectomy.

## Materials and Methods

This cross-sectional case-control study was performed from July 2020-2021. The purposive sampling was carried out to select all the men referred to a tertiary care center for head and neck surgery. They were diagnosed with laryngeal cancer and underwent a total laryngectomy. The control group was purposively selected from those referred for hematologic tests and routine checkups. All the subjects in the case and control groups (21 and 19 samples respectively) were heavy smokers with a mean age of 57.22±7.89 years who had been addicted to cigarette smoking for at least 5 years. The other main selection criteria were as follow: approximately equal socio-economic and educational level of all the participants, the absence of comorbidities, and no history of psychiatric treatments.

It is worth noting that the study protocol was approved by our ethical committee (ethical code: 

IR.SUMS.MED.REC.1400.16). Moreover, the participants were aware of the voluntary nature of their participation, the aim of the study, and the confidentiality of the data. The participants' sexual problems were identified using the international index of erectile function (IIEF) questionnaire. 

The case group was asked to complete the questionnaire five months after the total laryngectomy and its post-op follow-up, while the control group was encouraged to answer the questionnaire during routine medical references. This, to some extent, led to a longer sampling time despite the relatively small sample size.

The IIEF is an international self-administered 15-item Likert scale that assesses men's erectile functioning. The questionnaire has been culturally adapted and validated for Persian-speaking men (11). 

It addresses the five domains of erectile function, orgasmic function, sexual desire, intercourse satisfaction, and overall satisfaction. The severity of sexual dysfunction is determined based on the acquired scores that are explained below:

0-10: severe sexual dysfunction

11-16: Moderate sexual dysfunction

17-21: Moderate to mild sexual dysfunction

22-25: Mild sexual dysfunction

26-30: Normal sexual dysfunction

After data collection, statistical analysis was conducted using SPSS software (version 21). The evaluation of the group's differences in age distribution was performed through the independent sample t-test. 

In addition, a multivariate analysis of variance compared the group's five domains of erectile function mentioned before. 

Qualitative data were analyzed using the Pearson Chi-square test.

## Results

According to Table 1, which illustrates the participant's age distribution by independent one sample t-test, no significant difference is observed between the two groups.

**Table 1:** Group's age distribution

**Group**	**n**	**Mean±sd**	**Minimum**	**Maximum**	**t**	**df**	**Significance** **(P-value)**
Case	21	57.61±7.81	42	75	0.32	38	0.74
Control	19	56.78±8.17					

Also, 70%- 80% of patients in both groups had an education up to a high school diploma or lower. The Pearson Chi-square test showed no significant difference in the distribution of educational level of both groups (P≥0.05). The groups' mean scores in five sub-tests of IIEF are depicted in the table below.

**Table 2:** Mean scores of the groups in five sub-tests of the international index of erectile function questionnaire

**n**	**Standard Deviation**	**Mean**	**group**	**Sexual function**
21	9.03643	13.4286	Case	Erectile function
19	7.26362	19.2632	Control	
21	3.61215	5.0476	Case	Orgasmic function
19	2.43752	6.0526	Control	
21	2.55883	4.9524	Case	Sexual desire
19	2.26723	6.8421	Control	
21	4.16333	4.6667	Case	Intercourse satisfaction
19	3.93440	8.4211	Control	
21	2.72903	6.0476	Case	overall satisfaction
19	2.03622	6.5789	Control	

**Diagram 1:**
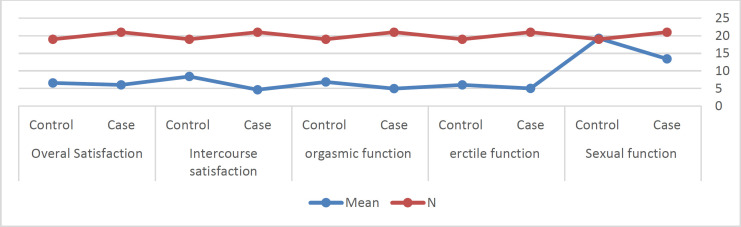
Comparison among groups in five sub-tests of the international index of erectile function questionnaire

As displayed in Table 2, the laryngectomy patient's mean scores in all five subtests of the IIEF scale are lower or approximately equal to that of the control group. In order to assess the significance of the difference between the two groups in terms of the five domains of erectile function, orgasmic function, sexual desire, intercourse satisfaction, and overall satisfaction, the multivariate analysis of variance was carried out, and the results are illustrated in Table 3 and Diagram 1.

**Table 3:** Test of between-subjects effects for five sub-tests of the international index of erectile function questionnaire in the intervention and control groups

**Source**	**Dependent variables**	**Type ш sum of square**	**df**	**Mean square**	**F**	**Significance**
**Group**	Erectile function	339.573	1	339.573	4.996	0.031
	Orgasmic function	10.075	1	10.075	1.041	0.314
	Sexual desire	35.621	1	35.621	6.057	0.019
	Intercourse satisfaction	140.602	1	140.602	8.545	0.006
	Overall satisfaction	2.816	1	2.816	.479	0.493

Based on Table 3, the laryngectomy patients performed significantly lower than the control group in three subtests of erectile function, sexual desire, and intercourse satisfaction (P<0.05). This pattern is also demonstrated in the below diagram.

## Discussion

It is confirmed that the quality of life in total laryngectomy patients is much worse than that in the normal population(1, 12). Among the factors studied regarding the quality of life, sexual functioning as an important factor greatly influenced by total laryngectomy needs to be considered (13). The present study, which was performed on 21 patients suffering from total laryngectomy, observed sexual problems, especially in the field of erectile function, sexual desire, and intercourse satisfaction. The unstructured interview performed about five months after the surgery indicated a sense of social isolation due to no verbal communication with others. This led to some degree of depression and sadness in total laryngectomy patients. The results of two different studies conducted in 2005 and 2010 indicated that permanent tracheostomy presents patients with various degrees of depression and sexual dysfunction in men (14, 15). It is confirmed that psychological features strongly affect sexual functioning in total laryngectomy patients. Loss of speech and communication disorders are the main predictors of anxiety and depression that might cause these patients to withdraw from others and live in a world of isolation due to hopelessness and low self-esteem in depressed patients(9,10,14,16).

A study in the Netherlands in 1995 was carried out on two groups of patients who suffered from laryngeal cancer (66 patients treated with radiotherapy alone and 32 patients experienced total laryngectomy). The results of the mentioned study indicated that difficulties, such as depression, anxiety, and sexual dysfunction, were significantly more serious in total laryngectomy patients. Moreover, women faced much more problems, especially in psychological adjustment, such as open communication about sexuality and their appearance (17). 

A study performed in 2008 depicted that more than half of the participants encountered a significant reduction in sexual enjoyment. However, only 60% of subjects considered it an important issue, and the rest acknowledged that speech and swallowing problems were more important (10). 

Apart from the remarkable results obtained from the present study, it has some limitations, such as a rather small sample size. In addition, there was no evidence of alcohol consumption in the participants. Furthermore, the participants were confined to male patients. That is why some factors, such as attraction and spouse's feelings about the quality of married life, which are so important to women, have not been considered. Therefore, the negative consequences of total laryngectomy need to be re-confirmed by assessing the vast majority of men and women who had experienced it. Finally, it is recommended to evaluate patients for problems with depression by standard depression Tests due to various degrees of depression discussed by them during the post-op interviews. 

## Conclusion

The quality of erectile function, sexual desire, and intercourse satisfaction of total laryngectomy men were significantly lower than that of their normal peers. Moreover, interviews with the patients five months after the surgery indicated a sense of social isolation, as well as some degree of depression and sadness, in total laryngectomy patients.
